# Efficacy of Anakinra on Multiple Coronary Arteries Aneurysms in an Infant with Recurrent Kawasaki Disease, Complicated by Macrophage Activation Syndrome

**DOI:** 10.3390/children9050672

**Published:** 2022-05-05

**Authors:** Grazia Bossi, Alessia Claudia Codazzi, Federica Vinci, Edoardo Clerici, Corrado Regalbuto, Carmela Crapanzano, Daniele Veraldi, Alice Moiraghi, Gian Luigi Marseglia

**Affiliations:** 1 Department of Pediatrics, Fondazione Istituto di Ricovero e Cura a Carattere Scientifico (IRCCS) Policlinico San Matteo, 27100 Pavia, Italy; 2 Pediatric Cardiology, Department of Pediatrics, Fondazione Istituto di Ricovero e Cura a Carattere Scientifico (IRCCS) Policlinico San Matteo, 27100 Pavia, Italy; a.codazzi@smatteo.pv.it; 3 Pediatric School of Specialization, University of Pavia, 27100 Pavia, Italy; fede90vinci@gmail.com (F.V.); ed.clerici88@gmail.com (E.C.); corrado.regalbuto01@universitadipavia.it (C.R.); carmela.crapanzano01@universitadipavia.it (C.C.); danieleveraldi92@gmail.com (D.V.); alice.moiraghi@gmail.com (A.M.); 4 Department of Clinical-Surgical Diagnostic and Pediatric Sciences, University of Pavia, 27100 Pavia, Italy; gl.marseglia@smatteo.pv.it

**Keywords:** Kawasaki disease, coronary aneurysms, interleukin-1, anakinra

## Abstract

Kawasaki disease (KD) is rare in infants less than 3 months of age, and its recurrence is exceptional. Infants with KD are at higher risk of severe clinical presentation, therapy failure, complications and coronary aneurysms (CAAs), and this is the reason they deserve more aggressive therapy and a strict clinical follow-up. We report a 2-month-old male with KD, complicated by Macrophage Activation Syndrome (MAS). Despite timely and aggressive therapy with immunoglobulins, steroids and aspirin, multiple CAAs developed. Two-month therapy with anakinra completely reverted all the aneurysms. After six months, the infant experienced KD relapse and was successfully re-treated with immunoglobulins, steroids and aspirin. A strict echocardiographic follow-up did not show recurrence of aneurysms. Two years later, the child is healthy, without cardiac sequelae. In our experience, anakinra was effective in reverting multiple aneurysms and its effect proved to be long-lasting, even in front of KD recurrence. Based on this evidence, it seems reasonable to hypothesize not to limit the use of anakinra as rescue therapy for complicated or refractory KD, but to consider the possibility of adding it to first-line therapies for some subgroups of very-high-risk patients, in order to strengthen the prevention of CAAs.

## 1. Background

Kawasaki disease (KD) is a rare, acute, inflammatory, multisystemic vasculitis, that mainly affects medium-sized vessels. Due to its selective tropism for coronary arteries, KD is the leading cause of acquired heart disease in developed countries [1,2]. Although its etiology is still unknown, KD is regarded as an immune-mediated disease that affects predisposed individuals upon exposure to unknown environmental triggers, likely infectious agents [3]. The role played by genetic factors is testified by the higher incidence of KD in patients of Asian ancestry and in first-degree family members [4].

Due to the lack of confirmatory laboratory tests, the diagnosis of KD is mainly clinical and still relies on the criteria published by the American Heart Association (AHA) in 2017: Fever, polymorphous rash, bilateral nonexudative conjunctivitis, oral mucosa lips erythema, peripheral extremities changes and cervical lymphadenopathy [2].

KD diagnosis can be more challenging when its presentation is characterized by fewer signs and symptoms (incomplete KD), or by very different features (atypical KD) [1].

KD mainly affects infants and children under 5 years of age and peaks in the first two years of life. Cases in infants under 3 months of age are exceptionally rare (1.6%) and are usually characterized by a higher prevalence of incomplete or atypical forms, delayed diagnosis, treatment failure and exceedingly higher risks of developing complications and coronary artery dilations or aneurysms (CAAs) [5,6]. One of the most severe complications of the acute phase of the disease is the Macrophage Activation Syndrome (MAS), which results from an uncontrolled immune response that generates excessive cytokines production, mainly interleukin-1β (IL-1β), interleukin-6 (IL-6), interleukin-18 (IL-18) and γ-interferon (γ-IFN) [7]. KD complicated by MAS requires more aggressive treatment, due to the higher risk of therapy failure, heart disease (46% vs. 25%) and death (13%) [8,9,10,11,12,13].

Recurrent KD is an exceptional event, most common in children of Asian ancestry, and is correlated to an exceptionally high risk of CAAs [14,15].

KD prognosis depends to a great extent on early diagnosis and prompt treatment with high-dose intravenous immunoglobulins (IVIG) and aspirin (ASA), which decreases the risk of CAAs from 20–25% to less than 5% [16].

Despite timely and proper treatment, 10–20% of patients develop fever again or have persistent fever 36 h after IVIG. Children with refractory KD are at a higher risk of developing CAAs [17,18]. This is the reason, in the most recent KD therapy protocols, such as the Italian one, first-line treatment intensification with glucocorticoids has been added to standard IVIG plus ASA for high-risk patients [19]. However, the real effectiveness of initial treatment intensification could be diminished by the lack of an effective scoring system for predicting the IVIG resistance in KD patients from western countries [20].

A large body of literature evidence from genetic studies and experimental KD mouse models strongly supports the pivotal role played by IL-1β in both KD pathogenesis and its cardiovascular lesions [21,22,23,24,25,26]. Anakinra, which competitively inhibits IL-1 binding to the IL-1 type 1 receptor, seems preferable over others (rilonacept and canakinumab) because of its good safety profile in pediatric patients, its rapid effect and short half-life and its ability to block both IL-1α and IL-1β. Based on this evidence, and given the success achieved by the IL-1 blockade in patients affected by cryopyrin-associated periodic syndrome (CAPS) and systemic-onset Juvenile Idiopathic Arthritis (JIA) [27,28], anakinra has been successfully employed in children affected by KD refractory to IVIG and severe cardiac complications [29,30,31,32,33,34].

Herein we report the case of a 2-month-old infant with classic KD, complicated by MAS. Despite timely and adequate treatment, the infant developed multiple CAAs. Therapy with anakinra proved to be safe and effective, leading to complete and stable regression of all CAAs. Six months after the first episode, the infant experienced a KD relapse and was successfully retreated, without recurrence of aneurysms. Two years after the disease relapse, the patient is healthy, with normal echocardiographic findings.

## 2. Case Presentation

**Figure 1:**
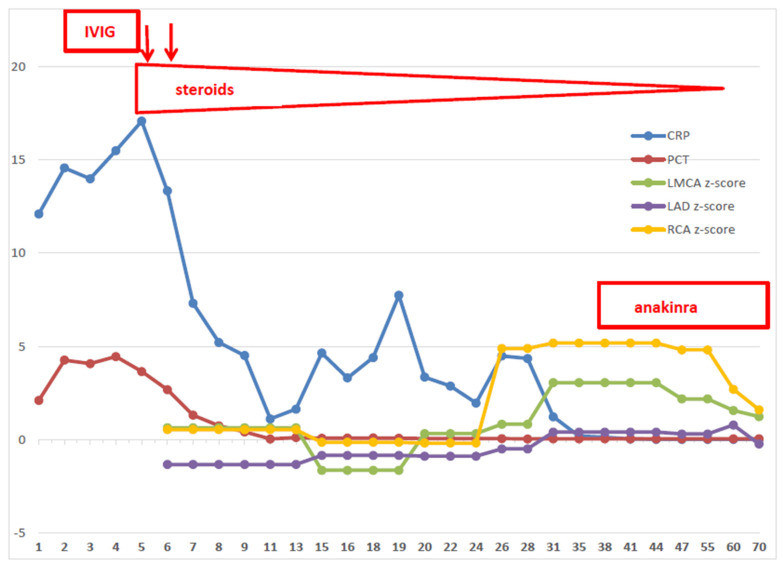
Time course of C-reactive protein, Procalcitonin, coronary arteries and treatments. CRP = C-reactive protein; PCT = procalcitonin; LMCA = left main coronary artery; LAD = left anterior descending artery; RCA = right coronary artery; IVIG = intravenous immunoglobulins.

A 2-month-old Caucasian male, second-born to healthy unrelated parents, was admitted to our department with a one-day history of high-picking fever, poor feeding and irritability. Laboratory tests showed leukocytosis (WBC 20.900/mm^3^) with neutrophilia (71%), normal hemoglobin (Hb) and platelet (PLT) values, elevated CRP (12.10 g/dL, n.v. < 0.5) and procalcitonin (PCT 2.1 ng/mL, n.v < 0.5). The chest X-ray revealed a left paracardiac parenchymal opacity. The nasal swab tested positive for Coronavirus 229E/NL63, but no other viruses (Epstein–Barr, Cytomegalovirus, Parvovirus B19, Adenovirus, Parmixovirus, Herpes Virus Type 6 and 7) could be detected by Polymerase Chain Reaction or serological tests; blood, urine and cerebrospinal fluid cultures were sterile. On day +3 from the fever onset, despite wide-spectrum antibiotic therapy, the infant was still highly febrile and, for respiratory distress requiring respiratory support, was referred to our NICU. On day +6, while the fever persisted, the clinical examination noted a diffuse reddish skin rash, associated with monolateral lymphadenopathy, bilateral nonexudative conjunctivitis, cheilitis and swollen feet. CRP and PCT had risen to 17 mg/dl and 3.65 ng/mL, respectively; a dramatic decrease in Hb values (7.4 g/dL) and PLT count (99 × 10^9^/L) was evident, together with high values of ferritin (1057 ng/mL), hypertriglyceridemia (228 mg/dL) and increased liver enzymes. Perforin expression and NK activity were normal. Thus, Kawasaki disease with concurrent MAS was diagnosed. The echocardiography did not show coronary abnormalities. According to the Italian Society of Pediatrics (SIP) KD guidelines for high-risk patients [19], the infant was promptly treated with IVIG (2 gr/kg), plus ASA at an anti-thrombotic dosage (5 mg/kg.day) and intravenous methylprednisolone (MPDN; 30 mg/kg.day, q8h). Due to fever persistence, the second dose of IVIG was administered on day +7, along with additional pulses of MPDN (10 mg/kg.day, q8h for 3 consecutive days). Shortly after the second pulse of IVIG, fever and all the other clinical signs subsided. WBC, Hb, PLT and ferritin values normalized; CRP progressively decreased. Then, MPDN was tapered and replaced by oral prednisone and the patient was strictly monitored with weekly echocardiographic evaluation. On day + 24, while the steroid therapy was still ongoing (1.5 mg/kg.day), the first aneurysm (z-score 4.89) became evident in the right coronary artery (RCA). Within a few days, a second aneurysm (z-score 5.18) was evident in the RCA, and the left coronary artery (LCA) become dilated. Clopidogrel (0.2 mg/kg/day) was promptly associated with ASA, and a total body angio-computed tomography was performed to rule out other aneurysms. Faced with such rapid evolution, with the aim to prevent further cardiac harm, and possibly revert the already established coronary damages, on day +35 we decided to start subcutaneous administration of anakinra (2 mg/kg.day), without stopping the ongoing tapering schedule of oral prednisone, clopidogrel and ASA. In the next weeks, the echocardiographic monitoring showed progressive z-score normalization, together with the impressive remodeling of both coronary arteries (Figure 1). Anakinra was administered for sixty-two days, with tapering doses of oral prednisone. The treatment was well-tolerated and was withdrawn, along with the dual antiplatelet therapy, when the complete and stable regression of all CAAs was evident. All the next clinical and echocardiographic controls confirmed the good condition of the patient and the normal size and shape of his coronary arteries.

Six months after the first episode, the patient (10 months old) was readmitted with clinical manifestations of classic KD, but without evidence of MAS. Compared to the most recent control, the coronary arteries did not show size or shape abnormalities. The infant was retreated according to SIP KD guidelines for high-risk patients: IVIG (2 doses), high-dose MPDN (3 pulses, then tapered) and anti-thrombotic dose of ASA. Due to the evidence of Influenza A infection (nasal swab), Oseltamivir (3 mg/kg.day, q12h) was added to therapy. The clinical response was good, with rapid disappearance of the fever and all other symptoms, and stable normalization of inflammatory markers and blood count. Two years after the second episode of KD, the baby is healthy, with normal echocardiogram and laboratory tests (Table 1).

## 3. Discussion and Conclusions

KD is very rare in the first three months of life, when the disease is often incomplete or atypical, and therefore at higher risk of treatment failure and complications [5,6]. MAS is one of the most severe, life-threatening complications of acute KD, and requires a very aggressive therapy [8,9,10,11,12,13]. Recurrence of KD is a very uncommon event, more often reported in children of Asian ancestry, and is associated with the risk of CAAs [14,15].

Our experience confirms all the above evidences from the literature. From August 2000 to April 2022, 90 children with KD have been diagnosed with KD at our center. Eighteen (20%) were younger than 12 months of age and only one (1%) was younger than 3 months. MAS occurred with certainty in three cases (3%) and KD relapse was diagnosed in three cases (3%).

Here, we reported the case of our youngest patient, a 2-month-old boy affected by KD, complicated by MAS, who developed multiple CAAs despite prompt and aggressive therapy with IVIG, aspirin and high-dose steroids. Treatment with anakinra turned out to be safe and effective in remodeling coronary arteries, with the disappearance of aneurysms. Coronary arteries retained their normal size and shape even when KD relapsed a few months later.

To the best of our knowledge, the current literature reports only one other case similar to ours: a 3-month infant affected by complete KD who, despite two courses of IVIG and corticosteroids, developed giant CAAs. Anakinra and prednisone were ineffective, and the aneurysms reverted only when a second biological (etanercept) was added [35]. However, the reported case differs from ours in some respects. Above all, at diagnosis, the patient was not classified as high risk according to the more recent risk-model scores adopted in western countries [36] and did not receive first-line corticosteroids together with IVIG. Furthermore, the anakinra therapy was initiated while the patient was still febrile, with elevated inflammatory biomarkers and when two giant CAAs had already developed. Based on this experience, it can be speculated that the biological agents have a short timeframe of action and that they could be effective in remodeling the coronary wall only before severe and irreversible damage (i.e., a high z-score) has occurred.

In our opinion, the case we presented here is unique in several respects and provides a valuable opportunity for some important insights.

Our patient, despite his very young age, had a classic form of KD, fully responsive to timely therapy with IVIG, ASA and high doses of steroids. Nonetheless, multiple CAAs developed more than 3 weeks after the disease onset, and worsened very rapidly, while the patient was steadily afebrile and still on steroid therapy. However, upon critically reconsidering the cardiac damage progression together with the pattern of inflammatory markers, it can be noted that the CAAs began to manifest and worsened while the CRP (not PCT) values, although significantly lower compared to disease onset, were not completely normalized. Our case proves that CRP values should be carefully monitored in KD patients, regardless of symptom improvement, because such a dichotomy between clinical recovery and CRP values can represent the biochemical issue of coronary artery damage still ongoing, despite fever disappearance and normal echocardiographic features. Therefore, the incomplete normalization of CRP values seems to have a predictive value of IVIG resistance higher than fever persistence or recurrence, and, in our opinion, should be considered among the parameters of every KD scoring system aimed to identify the patients that are candidates for more aggressive therapies.

Moreover, our experience testifies to the potential power of anakinra, not only to induce the full recovery of the aneurysms but also to induce a real remodeling of coronaries, stable even in cases of KD relapse. We acknowledge that the normal evolution of KD provides the possibility that moderately enlarged aneurysms can spontaneously regress. However, our patient developed small (z-score 2.5–5) and medium aneurysms (z-score > 5) despite a very aggressive first-line therapy, while the steroid treatment was still ongoing, and although it cannot be proved with certainty that the healing was fully attributable to anakinra, it is rather difficult to think that coronary diameters and shapes could have completely normalized without any therapy. What is instead undeniable is that the effect of anakinra on coronaries proved to be stable and long-lasting, despite KD recurrence a few months after treatment withdrawal.

In conclusion, our case confirms that anakinra could be very effective as a rescue treatment in patients with KD that develop CAAs, and that its remodeling activity is stable even in front of a truly rare event such as KD relapse.

Moreover, our experience also provides an opportunity for some other future issues.

The most recent KD guidelines pursue the goal of tailored treatment by the stratification of risk criteria and recommend the use of anakinra only as a rescue treatment for patients with complicated or resistant forms of KD that fail to respond to the second-line treatment. Unfortunately, the existing scoring systems for KD patients are not suitable for children other than of Asian ancestry [20]. As a consequence, in western countries, a subgroup of children at very high risk of cardiac complications still escape detection. In our experience, CRP seems to be a good predictive factor of therapy failure and is even more sensitive than fever persistence or recurrence.

Considering that most experimental studies and multiple case reports outline its successful clinical use, it seems reasonable to hypothesize not to limit the use of anakinra as rescue therapy for complicated or refractory Kawasaki disease, but instead, to consider the possibility of adding it to first-line therapies for some subgroups of very-high-risk patients in order to prevent heart complications in a more effective way. This suggestion gains more strength from the ongoing experience of clinicians increasingly facing the new challenges of Pediatric Inflammatory Multisystemic Syndrome (PIMS), closely related to KD and successfully treated with anakinra [37,38]. Whether these are distinct entities or different forms of the same clinical spectrum, it is unquestionable that hyperinflammation due to the cytokines storm is the hallmark of both these conditions.

## Figures and Tables

**Table 1:** Time course of CRP levels, coronary arteries diameters and z-score.

		ECOCARDIOGRAPHIC FINDINGS							
		RCA				LCA			
TIME	CRP mg/dl	PROXIMAL		DISTAL		MAIN		LAD	
		mm	z-score	mm	z-score	mm	z-score	mm	z-score
At diagnosis	17.08	1.5	0.53	1.3	−0.15	2	0.63	1.1	−1.34
+18 days after IVIG	4.4	2.8	4.89			2.1	0.82	1.3	−0.50
+35 days (anakinra start)	1.22	2.9	5.18	2.7	4.49	2.9	3.05	1.5	0.4
+30 days of anakinra	<0.01	1.9	1.6	2.5	3.64	2.3	1.23	1.4	−0.24
+120 days of anakinra	<0.01	1.3	−0.11	1.5	0.09	1.6	−1.07	1.3	−1.14
At relapse	19.47	1.7	0.38			1.5	−1.49	1.4	−0.89
+15 after IVIG	0.3	1.3	−0.87			1.7	−0.9	1.4	−0.83
+30 days	<0.01	1.4	−0.84			1.8	−0.63	1.5	−0.39
+6 months	<0.01	2	1.07			2.3	0.44	1.3	−1.58
+1 yr from relapse	<0.01	1.5	−0.6			2.0	0.5	1.2	−1.68
+2 yrs from relapse	<0.01	1.6	−0.35			2.2	0.6	0.9	−1.84

CRP: C-reactive protein; IVIG: intravenous immunoglobulin; RCA: right coronary artery; LCA: left coronary artery; LAD: left anterior descending.

## Data Availability

All the clinical data reported are available from the corresponding author on reasonable request.

## Abbreviations

KD	Kawasaki disease
AHA	American Heart Association
ESR	Erytrocyte Sedimentation Rate
CRP	C-Reactive Protein
IVIG	Intravenous Immunoglobulins
ASA	Aspirin
CAA	Coronary Artery Aneurysms
IL-1	Interlukin-1
MAS	Macrophage Activation Syndrome
US	United States of America
WBC	White Blood Cells
Hb	Haemoglobin
PLT	Platelets
LCR	Cerebrospinal fluid
SIP	Italian Society of Pediatrics
MPDN	Methylprednisolone
IL-1α	Interleukin-1 alfa
IL-1β	Interleukin-1 beta
PIMS	Pediatric Inflammatory Multisystemic Syndrome
